# A Social Group-Based Information-Motivation-Behavior Skill Intervention to Promote Acceptability and Adoption of Wearable Activity Trackers Among Middle-Aged and Older Adults: Cluster Randomized Controlled Trial

**DOI:** 10.2196/14969

**Published:** 2020-04-09

**Authors:** Jing Liao, Hai-Yan Xiao, Xue-Qi Li, Shu-Hua Sun, Shi-Xing Liu, Yung-Jen Yang, Dong (Roman) Xu

**Affiliations:** 1 Department of Medical Statistics & Epidemiology School of Public Health Sun Yat-sen University Guangzhou China; 2 Sun Yat-sen Global Health Institute School of Public Health and Institute of State Governance Sun Yat-sen University Guangzhou China; 3 Division of Health Management Shayuan Primary Health Care Center Guangzhou China; 4 Taiwanese Society of Geriatric Psychiatry Taiwan China

**Keywords:** mobile health, group exercise, social influence, behavior change, cluster randomized controlled trial

## Abstract

**Background:**

Wearable activity trackers offer potential to optimize behavior and support self-management. To assist older adults in benefiting from mobile technologies, theory-driven deployment strategies are needed to overcome personal, technological, and sociocontextual barriers in technology adoption.

**Objective:**

To test the effectiveness of a social group–based strategy to improve the acceptability and adoption of activity trackers by middle-aged and older adults.

**Methods:**

A cluster randomized controlled trial was conducted among 13 groups of middle-aged and older adults (≥45 years) performing group dancing (ie, square dancing) as a form of exercise in Guangzhou from November 2017 to October 2018. These dancing groups were randomized 1:1 into two arms, and both received wrist-worn activity trackers and instructions at the baseline face-to-face assessment. Based on the Information-Motivation-Behavior Skill framework, the intervention arm was also given a tutorial on the purpose of exercise monitoring (Information), encouraged to participate in exercise and share their exercise records with their dancing peers (Motivation), and were further assisted with the use of the activity tracker (Behavior Skill). We examined two process outcomes: acceptability evaluated by a 14-item questionnaire, and adoption assessed by the uploaded step count data. Intention-to-treat analysis was applied, with the treatment effects estimated by multilevel models.

**Results:**

All dancing groups were followed up for the postintervention reassessment, with 61/69 (88%) participants of the intervention arm (7 groups) and 56/80 (70%) participants of the control arm (6 groups). Participants’ sociodemographic characteristics (mean age 62 years, retired) and health status were comparable between the two arms, except the intervention arm had fewer female participants and lower cognitive test scores. Our intervention significantly increased the participants’ overall acceptability by 6.8 points (95% CI 2.2-11.4), mainly driven by promoted motivation (adjusted group difference 2.0, 95% CI 0.5-3.6), increased usefulness (adjusted group difference 2.5, 95% CI 0.9-4.1), and better perceived ease of use (adjusted group difference 1.2, 95% CI 0.1-2.4), whereas enjoyment and comfort were not increased (adjusted group difference 0.9, 95% CI –0.4-2.3). Higher adoption was also observed among participants in the intervention arm, who were twice as likely to have valid daily step account data than their controlled counterparts (adjusted incidence relative risk [IRR]=2.0, 95% CI 1.2-3.3). The average daily step counts (7803 vs 5653 steps/day for the intervention and control, respectively) were similar between the two arms (adjusted IRR=1.4, 95% CI 0.7-2.5).

**Conclusions:**

Our social group–based deployment strategy incorporating information, motivation, and behavior skill components effectively promoted acceptability and adoption of activity trackers among community-dwelling middle-aged and older adults. Future studies are needed to examine the long-term effectiveness and apply this social engagement strategy in other group settings or meeting places.

**Trial Registration:**

Chinese Clinical Trial Registry ChiCTR-IOC-17013185; https://tinyurl.com/vedwc7h.

## Introduction

The use of mobile technology in health care is becoming increasingly common [1], especially technologies that can support self-management [2]. By quantifying personal health data, mobile devices can facilitate behavior tracking and optimization [3]. Wearable activity trackers represent a subset of consumer mobile devices that can monitor physical activity and collect fitness-related data [4]. These trackers have gained popularity because of their affordable, nonintrusive, and useful features. In addition, recent reviews have suggested their effectiveness in promoting physical activity and improving health [5,6].

Despite their potential effectiveness, the voluntary use of activity trackers among older adults for health purposes remains limited. Compared to younger generations, middle-aged and older adults generally are less technology savvy [7] and continue to lag behind in technology adoption [8,9]. To date, three interrelated categories of barriers in technology adoption among older adults have been identified [10,11]. Person-related factors such as low literacy, limited income, poor health, and declined cognition have been associated with low technology acceptability [12] and adoption [9]. Technology-related factors, particularly perceived ease of use and usefulness, have been shown to largely explain variations in intention to use [13]. Finally, the social context under which technology is being used has also emerged as an important factor limiting adoption [13], such that the lack of assistance and a supporting environment may significantly reduce older adults’ intention and ability to use technology [10,14].

The complexity of older users’ reactions to mobile technology requires studies targeting improving technology acceptability and adoption in this population [8]. A few small-scale trials on activity tracker usage indicated that adults over the age of 50 years perceived their physical activity self-tracking experiences as acceptable and useful [15-17], particularly for fostering awareness of and motivation for physical activity [16,17]. Although most older adults held a positive attitude toward activity trackers, training and deployment strategies are still essential to overcome usage barriers [18]. Training on the core functions of activity trackers [15] combined with physical activity education [19] has been typically provided to older users as part of the deployment intervention, mainly targeting personal and technological barriers. Although crucial, training alone seems insufficient to meet the unique needs of older adults [19]. Given that activity trackers are less disease-oriented and require frequent user engagement [16], social influence and enjoyment components may be particularly relevant for their adoption [18]. A handful of recent studies examined the impact of sociocontextual drivers. By encouraging social support and comparison via face-to-face group discussions [20], online communications with virtual team members [21], or a combination of offline and online interactions [22], these studies found small yet significant increases in activity tracker usage. Despite these promising findings, two of these studies were not randomized controlled trials (RCTs) [21,23], and the only RCT pilot conducted to date was not properly designed to account for the social clustering effect [20]. Therefore, effective strategies for promoting technology acceptability and adoption among older adults remain elusive.

Considering this previous evidence, we adopted a social group–based deployment strategy and tested its effectiveness to improve the acceptability and adoption of activity trackers by middle-aged and older adults. The social group–based deployment strategy was developed based on the Information-Motivation-Behavioral Skills (IMBS) framework [23]. The IMBS framework specifies three components of behavioral change:

- Information: is directly related to the performance of the given behavior and permits cognitively effortless behavior-related decision making;
- Motivation: includes personal attitudes toward the outcomes (personal motivation) and perceived social norms for engaging in the behavior (social motivation);
- Behavior Skills: are essential for performing the behavioral change through enhancement of individuals’ skills and perceived self-efficacy.

We here report the results of phase 1 of a community-based cluster RCT (ChiCTR-IOC-17013185) to assess mobile technology–assisted interventions on the health of middle-aged and older adults. Mapping onto the IMBS framework, the phase 1 study aimed to address key personal, technological, and sociocontextual barriers simultaneously. We hypothesized that older adults who were facilitated by adequate information and behavior skills, and motivated by peers of their social groups would be more likely to accept and adopt wearable activity trackers than their counterparts exposed to these trackers independently.

## Methods

### Trial Design and Setting

This phase 1 report covers the trial conducted between November 2017 and October 2018, consisting of a 7-month recruitment and baseline assessment stage, a 3-month intervention stage, and the postintervention reassessment. Phase 2 focuses on health outcomes, whereas this phase 1 study prioritizes implementation outcomes. The study was carried out in Guangzhou, the capital city of Guangdong province, China. The trial development was guided by the CONSORT-eHEALTH Checklist [24] and CONSORT-Checklist for reporting a cluster RCT[25].

### Participants

In light of the fact that the social influences embedded in an existing social network can better motivate behavior change and adherence [26], the Chinese middle-aged and older adults who routinely practice dancing as a form of physical exercise on squares or other public spaces in groups (ie, square dancing) were chosen as our study population. Their main sociodemographic characteristics have been reported in a related study [27]. Briefly, major public squares and parks of three old districts of Guangzhou, namely Yuexiu, Haizhu, and Liwan, were identified via an online map (Baidu map), considering their land area, visitor flow, and residential locations. Using a restricted randomizing sampling approach, 8 squares and parks per district were chosen at random. We recruited participants in the selected squares and parks using advertisements and flyers. Square dancing groups regularly practicing in the selected places were used as the sampling frame. Dancing groups were eligible for the current study if the dancing style was not ballroom dance, and the total group size was no less than 20 with more than half of the dancers aged 45 years and older. Square dancers of the eligible dancing group were (1) community residents of Guangzhou, (2) regularly practiced square dancing at least once per week in the past 12 months, (3) aged 45 years and older, and (4) agreed to participate in our study if recruited. Participants were excluded if they (1) had serious and uncontrolled diseases related to the heart, brain, lung, liver, and kidney, or any acute complications; and (2) had no smartphone devices (as the data recorded by the wearable activity trackers can only be uploaded to the cloud via a paired smartphone device). Participants were initially screened for eligibility via onsite interviews, and eligible participants were invited for health checkups at the local community health centers on a scheduled date. All participants read and signed the written informed consent form approved by the Institutional Review Board (No. L2016-004) of the School of Public Health of Sun Yat-sen University.

### Random Allocation

All random allocation was performed at the cluster level, namely by the square dancing groups. After the recruitment of all eligible participants, a statistician otherwise not associated with the project allocated participants by their square dancing groups equally into two arms (1:1) following a simple randomization process. Although the participants were aware of the interventional nature of the study, they were blinded to their allocation status. Outcome assessors were blinded to the group assignments and were different from the researchers who conducted and monitored the interventions.

### Intervention and Procedures

#### Overview of Intervention

Both the intervention and control arms were equipped with wrist-worn activity trackers free of charge at the baseline assessment. Lifesense MAMBO2 wristbands [28] were used in this study, as their features were deemed to be suitable for our intervention. These activity trackers can automatically record and display participants’ step counts, heart rate, and exercising modes (eg, walking, dancing), and generate visualized daily physical activity reports on the paired smartphone via WeChat (the most popular communication and social media platform in China) or the Lifesense app available for ISO and Android systems. Participants’ WeChat IDs were linked with their Lifesense accounts to enable physical activity data sharing among peers. Individual participant data were securely aggregated and stored in the managerial account, which were then exported for analysis.

Participants in both arms received a 30-minute demonstration on the core functions of these activity trackers (eg, how to wear, read the displays, and charge the trackers), and were instructed to wear them throughout the day until going to bed. They were encouraged to ask questions and to test these functions during the tracker setup with the researchers. One project facilitator was assigned to assist one dancing group, who conducted the initial setups, provided troubleshooting, and monitored participants’ uploaded physical activity data via the managerial accounts.

**Figure 1:**
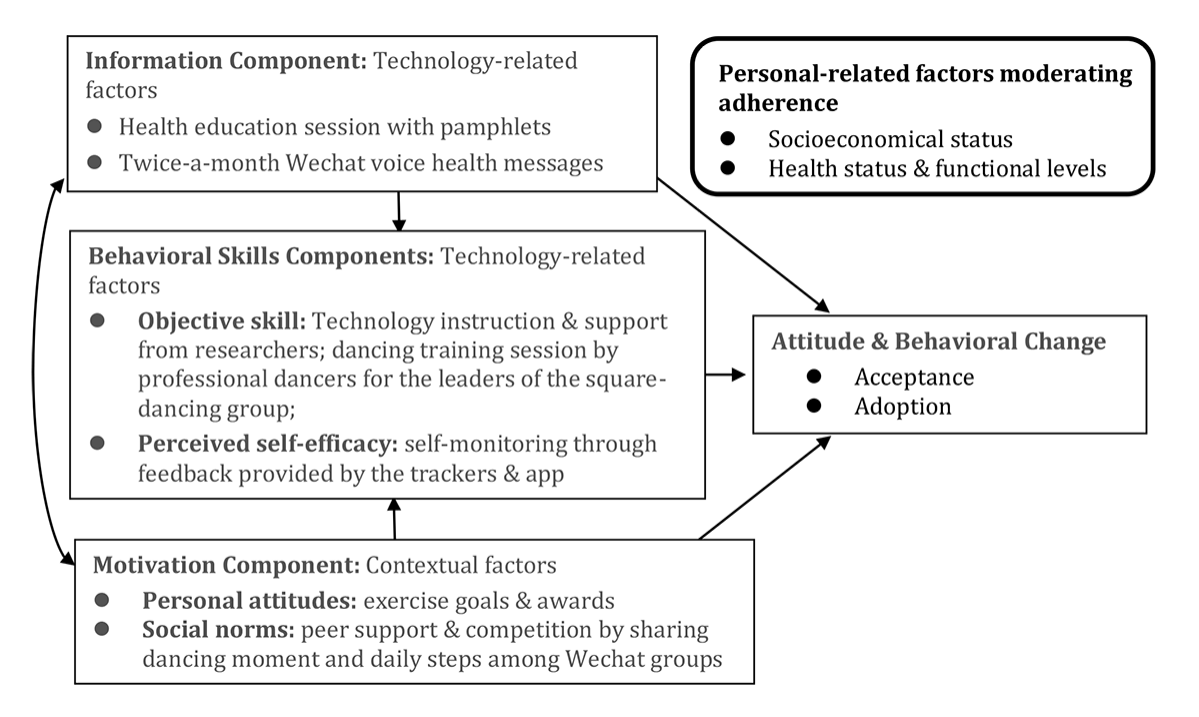
The Information-Motivation-Behavioral skills (IMBS) framework for intervention components.

The control arm received no other assistance, and these participants were left to use the activity trackers independently. The intervention arm was further assisted by the following three intervention components, grounded in the IMBS framework [23] (Figure 1).

#### Information Component

Educational information on the benefits of using activity trackers to monitor daily physical activity was delivered in two ways. The first involved a single 1-hour health education session on the recommended age-specific physical activity intensity, duration, and frequency. The participants also received a pamphlet with cartoon and bullet-point messages. The second involved six booster educational voice messages on physical activity-related topics such as warming up and relaxing exercises, which were delivered twice a month via the participants’ WeChat groups (defined further below). These messages consisted of a 1-minute voice message, along with the corresponding transcript and illustrations. The content of the education session and voice messages was designed based on the World Health Organization recommendations on physical activity for health [29] and the Chinese adult physical activity guideline [30].

#### Motivation Component

To motivate participants in using activity trackers for daily activity monitoring according to the physical activity recommendations, we teamed up participants of the same dancing group into their WeChat group, which was named after their dancing group. They were encouraged to share their daily physical activity progress recorded by the activity trackers with their peers in the WeChat group. At the end of each month, individual participants’ physical activity rankings identified by their WeChat nicknames were announced within their own group alongside their group’s overall physical activity ranking compared to that of other groups of the intervention arm. The participants and the groups with high rankings were awarded with corresponding points, which could be used to redeem gifts.

#### Behavior Skill Component

Skills on how to use these activity trackers for self-monitoring were delivered through the education sessions described above, assisted by our facilitators who provided in-time technology support and supervision. Additionally, a 2-hour dancing training session was delivered to the lead dancers of the intervention arm, regarding how to design their dancing exercises according to the physical activity recommendations with the assistance of the activity trackers. These lead dancers were identified as the pioneer of their own dancing group, who were able to reinforce behavioral change skills to their dancing peers.

### Outcomes

The current study focused on the process outcomes, namely the acceptability and adoption of wearable activity trackers.

Acceptability was defined as users’ subjective perception and experiences [6], and was evaluated by a 14-item user feedback questionnaire (Multimedia Appendix 1), adapted from a previous usability and acceptability study [15]. Rated on a 5-point Likert scale from 1 (“strongly disagree”) to 5 (“strongly agree”), this questionnaire assessed users’ acceptability in four main domains: enjoyment and comfort (3 items, range 3-15; Cronbach alpha=.85), motivation to use (4 items, range 4-20; Cronbach alpha=.83), usefulness (4 items, range 4-20; Cronbach alpha=.89), and perceived ease of use (3 items, range 3-15; Cronbach alpha=.76). A total score was calculated to indicate the users’ overall experience (Cronbach alpha=.93, range 14-70).

Adoption was defined as users’ interaction and usage behavior [6], and was evaluated objectively via the uploaded step count data in two ways: (1) the percentage of days with valid step records over individuals’ follow-up days (average 90.7 days), and (2) the average daily step counts per person of these valid step records. Daily step counts less than the 5th percentile of the study sample’s daily step counts (ie, 1311 steps per day) were treated as invalid records and were removed, as these steps might represent nonwear and inappropriate use of the activity trackers [31].

### Control Variables

Participants’ demographic characteristics (age, gender, marital status, children), socioeconomic status (education, retirement status, income), as well as self-reported health and any doctor-diagnosed chronic disease were assessed by a self-reported questionnaire. Health-related quality of life was examined by the Short Form Health Survey (SF)-12 [32], and cognitive function was evaluated by the Telephone Interview of Cognitive Status (TICS) and word recall tests, indicating participants’ executive function and short-term memory, respectively [33].

### Data Collection

Control variables were collected during the recruitment and baseline health checkup prior to the intervention by investigators and clinical staff who were blinded to the intervention assignment. During the intervention, data on participants’ daily physical activity level (eg, step counts) were automatically captured and uploaded by the wearable activity trackers and their paired smartphones as indicators for adoption. At the postintervention assessment, participants evaluated their satisfaction with the activity trackers by the 14-item questionnaire as an indicator for acceptability.

To further explore users’ experiences with the activity trackers, participants’ qualitative feedback was also collected by the group facilitators. In reference to the acceptability questionnaire, participants were encouraged to elaborate their self-monitoring experiences regarding enjoyment and comfort, motivation to use, usefulness, and ease of use. Their feedback was analyzed in a deductive manner to extract information concerning the barriers and facilitators of each acceptability domain. A formal coding process was not applied.

### Statistical Methods

The main study sample size was calculated based on changes in physical activity levels. This analysis showed that 12 square dancing groups with an average of 15 participants per arm would have 85% power to detect an increase in physical activity from 1302 to 1500 metabolic equivalent of task minutes per week, assuming an intracluster correlation coefficient (ICC) of 0.05 and a 5% type I error. To further account for a 20% attrition rate, 24 dancing groups across 3 districts were needed to fulfill a total sample size of 440 individual participants.

Descriptive analyses showing participants’ baseline characteristics for each arm were summarized at both the individual and cluster levels. The intention-to-treat (ITT) analysis was adopted to examine the treatment effects, minimize selection bias, and maintain the original randomization design [34]. Missing baseline covariates and missing outcomes were multiply imputed under the missing at random assumption, using individual demographic information, health status, and other outcomes and cluster identifiers, separately by randomized arms to avoid biasing treatment effects toward the null [35]. Altogether, 20 sets of complete datasets were imputed based on the chained equations. Primary analyses were then performed on each complete dataset, and combined results were obtained according to Rubin’s combination rules [36]. As participants were clustered within dance groups, multilevel linear regression models were used to test for the intervention effect on continuous outcomes (ie, acceptability) at the individual participant level, while taking the cluster-level variation due to dancing groups into account. Similarly, multilevel negative binomial models were fitted to count outcomes (ie, adoption), which followed an overdispersed Poisson-like distribution. All models were adjusted for baseline covariates that were empirically suggested to be strong predictors for the adoption of wearable trackers. The length of individual follow-up days was further adjusted in the multilevel negative binomial model for daily step counts. Sensitivity analyses were conducted among participants with complete cases. Analyses were conducted using STATA 15 (StataCorp, College Station, TX, USA).

## Results

### Participant Profile

**Figure 2:**
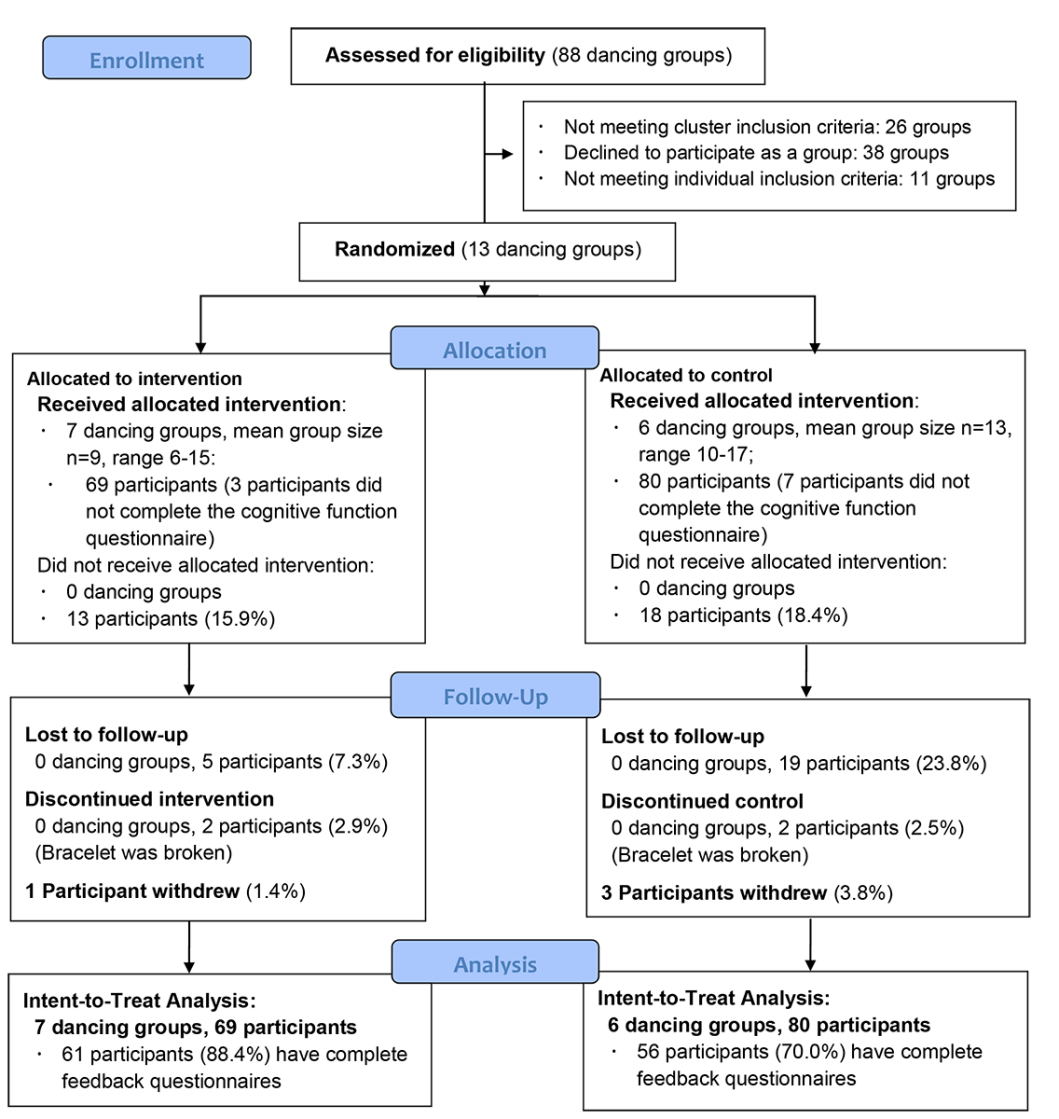
The flowchat of participants flow.

Figure 2 shows the participant flow. Of the 88 dancing groups initially assessed for eligibility, 26 groups did not meet the cluster inclusion criteria mainly because of dancing style and group size. Nearly half of the groups (38/88, 43%) assessed declined to participate due to lack of trust or time. The remaining 11 groups were also not eligible, as their group members were not local residents (n=2), had not been regularly practicing square dancing in the past 12 months (n=5), or less than 2 participants of the given group were willing to participate (n=4). The remaining 13 dancing groups were 1:1 randomized into the intervention arm (n=7) and the control arm (n=6). Among these eligible groups, 69 out of 82 (84%) participants of the intervention arm and 80 out of 98 (82%) participants of the control arm received the allocated treatment. During the follow up, no dancing groups withdrew; 5 participants of the intervention arm and 19 participants of the control arm were lost to follow up, 2 participants of each arm discontinued the intervention due to technical problems, and 1 participant of the intervention arm and 3 participants of the control arm withdrew from the study. The final ITT analysis sample was based on the 149 participants of 13 dancing groups, 117 (78.5%) of whom filled out the user feedback questionnaire of the wearable activity trackers.

Participants’ baseline characteristics are presented in Table 1. Sociodemographic characteristics and health status were similar between the two arms. Most of the participants were married older women (mean age 62 years), retired, with an education degree of senior high school; although some had been diagnosed with chronic diseases, all participants were physically and mentally sound. The intervention arm had fewer female participants and lower mean TICS scores than the control arm. The average group size was 9.9 and 13.3 participants for the intervention arm and the control arm, respectively.

**Table 1:** Participant characteristics at baseline by intervention and control arms (N=149)^a^.

Variables		Intervention arm (n=69)	Control arm (n=80)
Age (mean, SD)		61.8, 5.6	62.0, 5.0
Female (n, %)		63, 91	80, 100
**Marital status (n, %)**			
	Married	60, 87	69, 86
	Divorced	1, 1	3, 4
	Widowed	6, 9	6, 8
	Single	2, 3	2, 3
Having children (n, %)		67, 97	77, 96
**Education degree (n, %)**			
	Primary school and below	6, 9	2, 3
	Junior high school	14, 20	14, 18
	Senior high school	38, 55	52. 65
	University and above	11, 15	12, 15
Retired (n, %)		65, 94	80, 100
**Income^b^ (n, %)**			
	<10,000 yuan	15, 25	7, 13
	10,000 to <30,000 yuan	20, 32	16, 29
	30000 to <50,000 yuan	23, 38	26, 46
	50,000 to <10,000 yuan	3, 5	6, 11
	≥100,000 yuan	0, 0	1, 2
**Self-reported health (n, %)**			
	Very good	4, 6	9, 11
	Good	34, 49	34, 43
	Average	30, 43	34, 43
	Bad	1, 1	3, 4
	Very bad	0, 0	0, 0
Diagnosed chronic disease (n, %)		32, 46	48, 60
**SF-12^c^ scores (mean, SD)**			
	Physical Health Score	47.1, 7.7	46.3, 7.5
	Mental Health Score	53.5, 8.3	53.1, 8.6
	TICS^e^ score	8.2, 2.0	8.9, 1.5
	Word recall test score	4.7, 1.9	4.5, 1.6
**Cluster level (mean, SD)**			
	Number of dancing groups	7	6
	Number of participants per group	9.9, 3.1	13.3, 2.5

^a^Baseline descriptions were based on unadjusted raw data without imputations.

^b^Missing values at baseline were income (n=117) and cognitive function scores (n=139).

^c^SF-12: Short Form Health Survey-12; divided into physical health and mental health scores, ranging from 0 to 100, where a score of 0 indicates the lowest level of heath, and 100 indicates the highest level of health.

^d^Cognitive function was evaluated by the TICS and word recall tests scored from 0 to 10, where higher scores indicate better function.

^e^TICS: Telephone Interview of Cognitive Status.

### Outcomes Evaluation

Table 2 presents the unadjusted distributions of acceptability and adoption outcomes at the postintervention assessment in the intervention and control arms, along with the adjusted group differences for acceptability outcomes and the relative risks for adoption outcomes. A clustering effect was observed in most of the measures. The ICC ranged from 0.01 to 0.17 of the acceptability outcomes, and the between-group variance of the adoption outcomes was 0.24.

**Figure 3:**
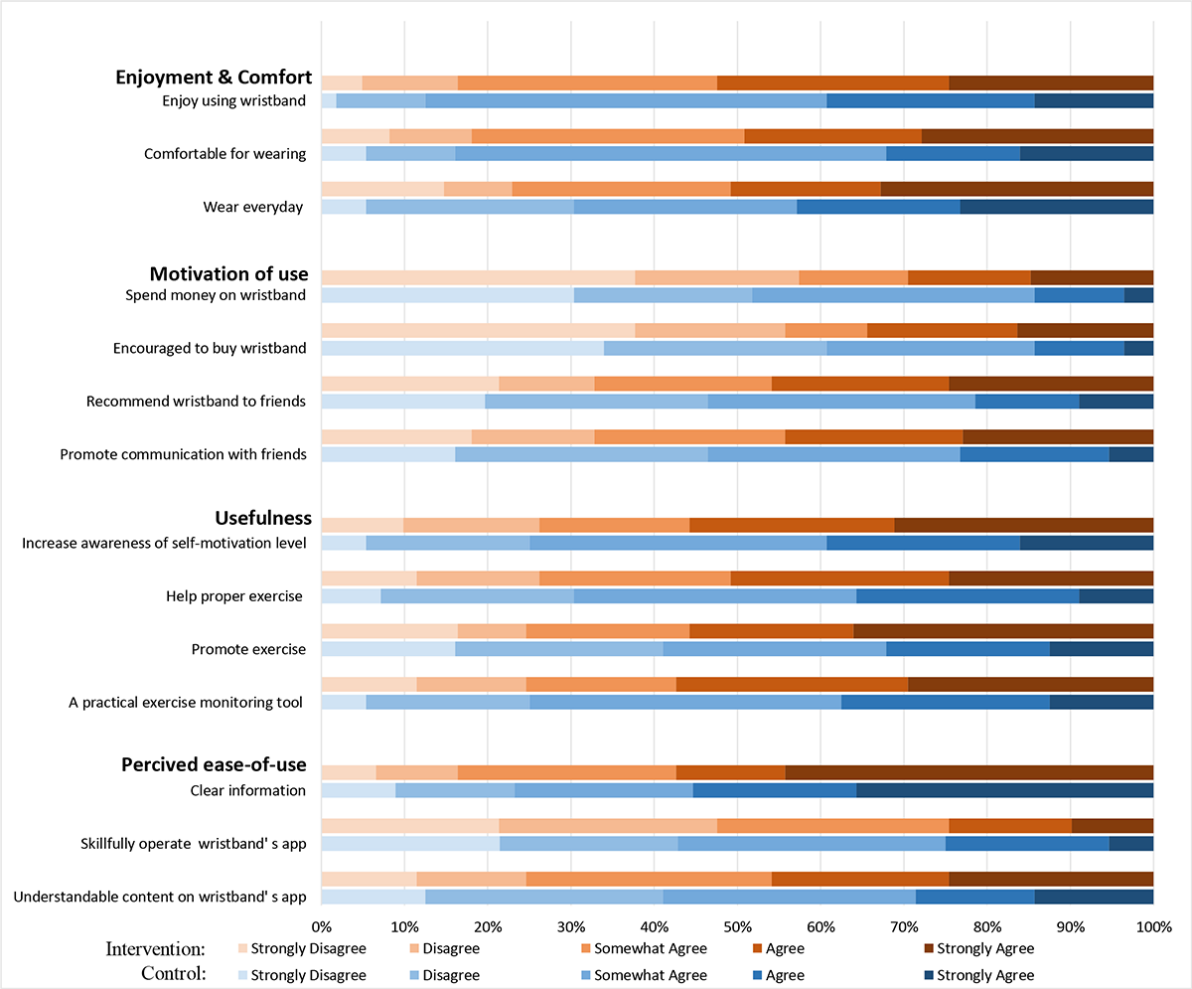
Indiviudal item rating for acceptability questionnaire by intervetion arms.

The unadjusted mean overall acceptability score (range 14–70) was higher for the intervention arm than for the control arm (Table 2). Raw ratings of individual items in each arm are shown in Figure 3. After adjusting for the clustering effect, baseline unbalanced covariates, and predictors for adoption, the absolute group difference in the overall acceptability score was estimated to be higher in the intervention arm than in the control arm. Examination on subdomains of acceptability further indicated that the difference in the overall score was mainly driven by promoted motivation, increased usefulness, and better perceived ease of use, but not due to enjoyment and comfort of using the activity trackers (Table 2).

Regarding adoption outcomes measured by step count data, the median percentage of days that participants had a valid step count record was 44.1% and 11.4% for the intervention and control arms, with 10% and 25% of each arm having invalid step counts records, respectively. As estimated by the multilevel negative binomial models, participants of the intervention arm were twice more likely to have valid daily step count data than their controlled counterparts. The average daily step counts were higher for the intervention arm than for the control arm, but the difference was not significant (Table 2). Similar findings were obtained among participants with complete cases and all covariates in the sensitivity analyses (Multimedia Appendix 2).

**Table 2:** Participant acceptability and adoption with activity trackers according to intervention status (N=149)^a^.

Variables		Intervention Arm (n=69)	Control Arm (n=80)	ICC^b^/ Var-_Group_^c,d^	Adjusted group difference (incidence relative risk and 95% CI)^e^
**Acceptability^f^, mean (SE)**					
	Overall Acceptability	45.2 (2.9)	40.4 (1.3)	0.12	6.8 (2.2-11.4)
	Enjoyment and Comfort	10.4 (0.6)	9.7 (0.5)	0.09	0.9 (–0.4-2.3)
	Motivation of use	11.1 (0.8)	9.7 (0.5)	0.01	2.0 (0.5-3.6)
	Usefulness	13.6 (1.1)	12.1 (0.4)	0.17	2.5 (0.9-4.1)
	Perceived ease-of-use	9.6 (0.5)	8.9 (0.5)	0.05	1.2 (0.1-2.4)
**Adoption, median (25%-75% IQR^g^)**					
	Percentage of days with step counts (%)	44.1 (5.4-84.4)	11.4 (0.5-36.7)	0.24	2.0 (1.2-3.3)
	Average daily step count (steps/day)	7803 (5683-9724)	5653 (1052-8462)	1.09E-26	1.4 (0.7-2.5)

^a^Estimates represent the combined results of 20 sets of complete datasets imputed by the chained equations.

^b^ICC: intracluster correlation coefficient.

^c^Var-_Group_: between-group variance.

^d^Group comparison models were adjusted for age, gender, education degree, income, SF-12 physical and mental health scores, and cognitive scores measured at baseline; group differences in acceptability outcomes were estimated by multilevel linear regression models.

^e^Incidence relative rate of the adoption outcomes between groups was estimated by multilevel negative binomial models.

^f^Acceptability was evaluated by a 14-item user feedback questionnaire on a 5-point Likert scale from 1 (“strongly disagree”) to 5 (“strongly agree”). The overall acceptability scores ranged from 14 to 70, comprising 4 subdomains: users’ enjoyment and comfort (3 items, range 3-15), motivation of use (4 items, range 4-20), usefulness (4 items, range 4-20), and perceived ease of use (3 items, range 3-15).

^g^IQR: interquartile range.

### Qualitative Feedback From Participants and Observations From Researchers

Qualitative feedback from participants and researchers’ observations indicated some common problems encountered by both arms. In terms of enjoyment and comfort of wearing, participants noted that the activity trackers were extremely uncomfortable to wear when the weather was hot and humid, let alone during dancing if they were sweating heavily. Some participants reflected that the figures displayed on the trackers were too small to read, such that they had to wear glasses to read them. Regarding usefulness, a few participants thought that the function of the wearable activity trackers was quite limited, whereas other functions such as blood pressure monitoring were considered to be more useful and relevant from their point of view. Some participants also commented that they preferred the sleep monitoring function to the physical activity monitoring function. As for ease of use, our researchers found that they most frequently received inquiries about how to charge the trackers and how to synchronize data with the paired smartphone.

## Discussion

### Principal Findings

We assessed the effectiveness of a social group–based deployment intervention informed by the IMBS framework to promote the acceptability and adoption of wearable activity trackers among community-dwelling middle-aged and older adults. In line with our hypotheses, the results revealed that our intervention significantly increased participants’ acceptability, mainly driven by improvements in the perceived motivation, usefulness, and ease of use. The intervention also effectively promoted participants’ adoption, quantified as twice the amount of valid step count data of the intervention arm than the control arm.

In terms of acceptability, we found that the participants (age range 47-75 years) perceived their self-tracking experiences positively, consistent with findings among Western adults over the age of 50 years [15-18]. Prior trials identified lack of awareness about activity trackers [18] and lack of support while using them (eg, setup, charge battery, and data interpretation) [16,17] as the main use barriers for older adults. Moreover, social support and social learning from a peer [37] were also suggested as key factors to sustain long-term use [15]. In view of previous findings, our deployment strategy specifically included a tutorial on the activity trackers’ functions and their value for participants (Information Component), provided technology support (Behavioral Skill Component), and encouraged social support and comparison within/between groups. Our intervention’s effectiveness was demonstrated by the promoted overall acceptability of the intervention arm over the control arm. The three subdomains of acceptability, namely usefulness, ease of use, and motivation, showed an average increase of 2 points postintervention, which may be largely attributed to the corresponding intervention components. However, our intervention did not alter the participants’ perceived enjoyment and comfort in using activity trackers. It is suspected that the burden of sharing and synchronizing physical activity data, and the physical discomfort of wearing activity trackers during the humid summer (ie, our intervention period) may have negatively affected their user experiences.

The effectiveness of our deployment strategy was also supported by the adoption data, such that the intervention arm had more frequent interactions with and valid use of the activity trackers than the control arm. These findings agree with prior social connectivity–enhanced trials with device-generated outcomes among older adults. McMahon and colleagues [20] reported that physical activity education delivered to small groups facilitated by in-class discussion and experience sharing (ie, interpersonal strategy) was more effective to promote physical activity than intrapersonal strategies (eg, personal goal setting). Lyons and colleagues [21] assigned participants into premade virtual teams to allow anonymous “likes” and comments, and found small increases in daily walking time and step counts. Butry and colleagues [22] combined both group sessions and online community boards to encourage physical activity assisted by activity trackers, and found frequent tracker usage and increased physical activity that was well maintained over the 6-month follow up. Our social group–based deployment strategy was only supported by the percentage of valid step count data, but not the average daily step counts. Similar daily step counts between the intervention and control arms may reflect a ceiling effect among square dancers, who were already physically active at baseline and were less likely to be more active over a short-time period.

### Strengths and Limitations

Our study contributes to the literature of the technology acceptance model (TAM) [38] owing to its rigorous RCT design, and the utilization of existing social networks to foster the acceptance and adoption of activity trackers. The TAM, originating from the field of psychology [39], posits the perceived usefulness and ease-of-use as the only drivers of usage intention and behavior. Although theoretical extensions on the basic TAM constructs have been suggested over the years, the majority of these studies are surveys or single-arm trials without appropriate comparisons, and the effect of sociocontextual modifications on technology acceptance is not well understood [38]. The extent to which older adults would like to interact with unfamiliar individuals in their age group may be quite different from their existing social network [26] where privacy would be less of a concern [17] and constant support is guaranteed. We deliberately applied our intervention among amateur Chinese square dancing groups, leveraging their group dancing routine and frequent interactions to enhance the social influence on their activity tracker-use behaviors. Despite the promising effect identified, we note that our participants were mostly females and middle-aged or slightly older, and thus extrapolating the implication to general older adults should be exercised with caution. Our empirical study nevertheless may inform further investigations on mobilizing community groups and social networks to promote voluntary technology usage among older adults.

Several limitations of our study are worth noting. First, significantly underestimating the challenges in the recruitment, we recruited only 13 dancing groups rather than the 24 groups planned. The participants recruited were thus more likely to be prone to using technology than general middle-aged and older adults. Nevertheless, this self-selection of the participants should not affect the internal validity of the effects of our program, as the intervention and control arms shared similar characteristics and were randomly assigned. However, the self-selection limited our program implication to the broader middle-aged and older population. Second, as our intervention package addressed the three main barriers of behavior changes jointly according to the IMBS framework, we were not able to distinguish the unique contribution of each intervention component to the program effect. Additional research designs may be considered in future studies, such as a factorial experiment [20] that may allow for evaluation of sole and joint effects of such an intervention. Third, our intervention relied on social interactions and required regular assistance from the research staff, particularly in the initial phase. Although social support and comparison functions have been integrated into many activity trackers recently [40], our assistance level is likely to be higher than that typically provided by commercially available activity trackers [16]. Lastly, as a phase 1 study, we have yet to capture and report long-term acceptability, adoption, and health-related outcomes, which are needed to establish the intervention’s long-term behavior maintenance and effectiveness.

### Conclusion

To ensure that the older population can benefit from mobile technologies, effective deployment strategies to promote technology acceptability and adoption are needed. We applied a social group–based intervention to address personal, technological, and sociocontextual usage barriers to amateur square dancing groups, and found improved acceptability and adoption of activity trackers among middle-aged and older square dancers who were mostly female. Our findings warrant future studies to investigate this social engagement strategy in other group settings of an existing social structure or meeting places, especially among less active male older adults.

## Appendix

Multimedia Appendix 1 Acceptability questionnaire.

Multimedia Appendix 2 Participants’ acceptability and adoption with activity trackers according to intervention status with complete cases.

Multimedia Appendix 3 CONSORT-eHEALTH checklist (V 1.6.1).

## Abbreviations

ICC: intracluster correlation coefficient
IMBS: Information-Motivation-Behavioral Skills
IQR: interquartile range
IRR: incidence relative risk
ITT: intention to treat
RCT: randomized controlled trial
SF: Short Form Health Survey
TAM: technology acceptance model
TICS: Telephone Interview of Cognitive Status
