# Supplementary material for: A Social Group-Based Information-Motivation-Behavior Skill Intervention to Promote Acceptability and Adoption of Wearable Activity Trackers Among Middle-Aged and Older Adults: Cluster Randomized Controlled Trial
Source: JMIR Mhealth Uhealth. 2020 Apr 9;8(4):e14969. doi: 10.2196/14969 (PMC7180511; doi:10.2196/14969)
Supplement: Multimedia Appendix 1 [file mhealth_v8i4e14969_app1.docx]

| **Question** | **Response** | | | | |
| --- | --- | --- | --- | --- | --- |
|  | **Strongly**  **disagree** |  | | | **Strongly**  **agree** |
| **Enjoyment & Comfort** | **1** | **2** | **3** | **4** | **5** |
| 1. Overall, do you enjoy using the wristband? | **1** | **2** | **3** | **4** | **5** |
| 2. Is the wristband comfortable to wear? | **1** | **2** | **3** | **4** | **5** |
| 3. Would you like to use the wristband every day? | **1** | **2** | **3** | **4** | **5** |
| **Motivation of use** | **1** | **2** | **3** | **4** | **5** |
| 4. Will you buy a sports wristband? | **1** | **2** | **3** | **4** | **5** |
| 5. If people around you using wristbands, will you be encouraged to buy one? | **1** | **2** | **3** | **4** | **5** |
| 6. Will you recommend wristbands to your friends? | **1** | **2** | **3** | **4** | **5** |
| 7. Will using wristband improve communication between you and your dance-mates? | **1** | **2** | **3** | **4** | **5** |
| **Usefulness** | **1** | **2** | **3** | **4** | **5** |
| 8. Did data generated by the wristband help you be aware of your exercise status? | **1** | **2** | **3** | **4** | **5** |
| 9. Did the wristband help you exercise properly? | **1** | **2** | **3** | **4** | **5** |
| 10. Did sharing exercise information motivate you to exercise? | **1** | **2** | **3** | **4** | **5** |
| 11. Generally, do you think the wristband is a practical exercise monitor? | **1** | **2** | **3** | **4** | **5** |
| **Perceived ease-of use** | **1** | **2** | **3** | **4** | **5** |
| 12. Is the information shown on wristband clear? | **1** | **2** | **3** | **4** | **5** |
| 13. Can you navigate the wristband app easily? | **1** | **2** | **3** | **4** | **5** |
| 14. Is the content provided by the wristband app easy to understand? | **1** | **2** | **3** | **4** | **5** |

**Multimedia Appendix 1. Acceptability questionnaire.**
