# Supplementary material for: A Social Group-Based Information-Motivation-Behavior Skill Intervention to Promote Acceptability and Adoption of Wearable Activity Trackers Among Middle-Aged and Older Adults: Cluster Randomized Controlled Trial
Source: JMIR Mhealth Uhealth. 2020 Apr 9;8(4):e14969. doi: 10.2196/14969 (PMC7180511; doi:10.2196/14969)
Supplement: Multimedia Appendix 2 [file mhealth_v8i4e14969_app2.docx]

**Multimedia Appendix 2: Participants acceptability and adoption with activity trackers by intervention status with complete cases.** ^a^

| Variables | **Intervention Arm** | | **Control Arm** | | ICC / Var-_Group_ | Adjusted Group Difference /Incidence Relative Risk ^b^  with 95%CI | | |
| --- | --- | --- | --- | --- | --- | --- | --- | --- |
|  | Mean /Medium ^a^ | SD /25%, 75% | Mean /Medium ^a^ | SD /25%, 75% |  |  |  |  |
| **Acceptability** ^c^ |  |  |  |  |  |  |  | |
| **Overall Acceptability** | 45.6 | 14.6 | 41.4 | 10.6 | 0.18 | 6.2 | 1.1 | 11.4 |
| Enjoyment & Comfort | 10.5 | 3.3 | 10.0 | 2.8 | 0.13 | 0.7 | -0.6 | 2.1 |
| Motivation of use | 11.4 | 5.0 | 9.9 | 3.5 | 0.06 | 2.0 | 0.5 | 3.6 |
| Usefulness | 13.9 | 4.9 | 12.4 | 3.7 | 0.25 | 2.2 | 0.4 | 4.0 |
| Perceived ease-of-use | 9.8 | 3.2 | 9.1 | 3.1 | 0.16 | 1.2 | 0.1 | 2.4 |
| **Adherence** |  |  |  |  |  |  |  | |
| Percentage of days with step counts (%) | 44.1 | 5.4, 84.4 | 11.4 | 0.5, 36.7 | 0.24 | 1.7 | 1.1 | 2.8 |
| Average daily step count (steps/day) | 7803 | 5683, 9724 | 5653 | 1052, 8462 | 1.57E-24 | 1.2 | 0.7 | 2.1 |

SD: standard deviation; ICC: Intracluster correlation coefficient; CI: confidence interval; Var-_Group_: between group variance.

^a.^ The complete case analysis sample size was 115 for acceptability outcomes and 139 for adhere outcomes. For acceptability, mean, SD and ICC were reported; while the adherence were described by medium, 25%~75% and between group variance.

^b^ Group comparison models were adjusted for age, gender, education degree, income, SF-12 physical and mental health scores and cognitive scores measured at baseline. Group differences in satisfaction outcomes were estimated by multilevel linear regression models. Incidence relative rate of the adherence outcomes between groups were estimated by multilevel negative binomial models.

^C^ Acceptability was evaluated by a 14-item users’ feedback questionnaire on a five-point Likert scale from 1‘strongly disagree’ to 5 ‘strongly agree’. The overall acceptability scores range from 14 to 70, which were comprised of four subdomains, namely, users’ enjoyment and comfort (three items, range 3~15), motivation of use (four times, range 4~20), usefulness (four items, range 4~20) and perceived ease-of-use (three items, range 3~15).
